# Integrative natural health solutions for midlife women

**DOI:** 10.3389/fnut.2025.1629648

**Published:** 2025-09-24

**Authors:** Vanita Dahia, Will Shannon, Anastasia Suraev, Sandra Villella, Gerald Münch, Diana Chan-Lau, David J. Hunter

**Affiliations:** ^1^Empowered Health, Blackburn, VIC, Australia; ^2^NutriPATH Pathology, Burwood, VIC, Australia; ^3^Pinnacle Health, Sydney, NSW, Australia; ^4^Healthy Brain Ageing Program, Brain and Mind Centre, School of Psychology, Faculty of Science, University of Sydney, Camperdown, NSW, Australia; ^5^Jean Hailes for Women’s Health, Melbourne, VIC, Australia; ^6^Torrens University, Melbourne, VIC, Australia; ^7^Pharmacology Unit, School of Medicine, Western Sydney University, Campbelltown, NSW, Australia; ^8^Medical Affairs, Haleon Pty Ltd, Parramatta, NSW, Australia; ^9^Sydney Musculoskeletal Health, Faculty of Medicine and Health, University of Sydney, Sydney, NSW, Australia; ^10^Rheumatology Department, Royal North Shore Hospital, Sydney, NSW, Australia

**Keywords:** complementary medicine, evidence-based medicine, middle-aged, women, health

## Abstract

Global interest in natural health products (NHPs) as complementary or alternative treatments is growing, especially among midlife women, due to their diverse health needs. Despite increased NHP use and benefits suggested by traditional use, the available scientific evidence supporting NHP efficacy and safety is often inconsistent, leading to hesitancy among health professionals about their use, even when reasonable evidence exists. Here, we offer a multidisciplinary perspective on optimizing NHP use alongside conventional clinical management in midlife women’s health. We advocate for robust systematic frameworks to generate and evaluate evidence from well-designed clinical studies, employing methodologies like those used for conventional medicines, but adapted to address the unique complexities of NHPs. We highlight key considerations for designing and developing NHP formulations: understanding differences in the activity spectrum of distinct extracts, the bioavailability of active compounds, and the chemical forms of the products. We also emphasize the importance of effectively communicating evidence on NHPs and its implications to health professionals and consumers, so as to maximize therapeutic benefits and minimize potential health risks. Essential actions include incorporating NHP education into continuing education programs for health professionals and enhancing public health literacy to promote safe, responsible, and informed use among consumers. Evidence-based approaches and multidisciplinary collaborations will help drive these essential actions and accelerate the complementary use of NHPs in improving health outcomes.

## 1 Introduction

Natural health products (NHPs)—including herbal medicines, vitamins, and mineral supplements—are known by various names worldwide, such as dietary supplements in the United States, complementary medicines in Australia, and botanicals and/or nutraceuticals among naturopaths. The term encompasses a wide range of products marketed as *“supplements”* to enhance health and/or as *“medicines”* to treat illnesses, but remains largely outside conventional medical practice (1–3). This variability reflects how different jurisdictions around the world regulate and classify NHPs. Some jurisdictions treat them under food-related guidelines, while others regulate them as medicines (1). For example, the Australian health authority recognizes natural and complementary medicines as substances with therapeutic effects rather than merely supplements (1, 4).

Over the past two decades, global consumption of NHPs has steadily increased, with unprecedented growth in market size and product diversity (1, 3). The increased relevance of NHPs is also reflected in Australia’s contemporary health and wellness landscape, with nearly half of Australians (49.4%) reporting the use of NHPs in 2022 (3, 5). Growth in consumer demand for NHPs has been driven by expectations of their benefits, dissatisfaction with conventional medical options, greater perceived safety of “natural” options, and the desire for greater control over healthcare choices (6, 7). This shift is particularly notable among middle-aged and older adults, individuals with chronic conditions, and women (5, 7, 8).

For many women, midlife represents a challenging period, marked by significant physiological transitions, such as menopause, and the onset of diseases that profoundly impact health and quality of life (QoL) (9–12). For example, the onset of vasomotor symptoms (VMS), such as hot flushes (flashes) and night sweats, can severely reduce QoL by causing sleep disturbances, fatigue, and mood swings (11, 13–15). During midlife, many women also experience changes in body composition, including increased visceral adiposity and a shift in fat distribution toward the middle section of the body (16, 17). Changes in sex hormones, body fat distribution, and lipid profiles, as well as sleep disturbances, can increase the risk of conditions like obesity, sarcopenia, osteoporosis, and cardiovascular disease (CVD) (17–21). Chronic pain is another significant issue, and studies indicate that women experiencing a higher burden of menopause symptoms are nearly twice as likely to suffer from chronic pain than those with fewer or no symptoms (22). Mobility issues and an increased prevalence of disabilities also become more common during midlife, often linked to obesity, arthritis, and other chronic conditions (20). Mental health is another important area, as perimenopausal and postmenopausal women are at higher risk for developing depressive symptoms and major depressive disorder compared with premenopausal women (23–25). Moreover, cognitive complaints, such as memory problems, are more common among midlife women, potentially attributable to hormonal fluctuations observed during this period, compounded by age-related decline in cognitive performance (26, 27).

Conventionally, management of women’s health in midlife has focused on Menopausal Hormone Therapy (MHT), also known as Hormone Replacement Therapy (HRT) (15, 28). This approach, which is based on estrogen, progesterone, or their combinations, is an effective way to control menopausal symptoms while also providing other health benefits (15, 28). Other issues with broad health implications, such as sleep or pain, are often addressed symptomatically and in isolation, rather than holistically through combined medical, behavioral, environmental, and motivational approaches (15, 29, 30). Misconceptions about MHT use, such as those related to outdated evidence from a 2002 study suggesting that MHT use increases the risk of breast cancer, may lead many midlife women to seek alternative approaches for their menopausal symptoms (31). Placing greater value on personal agency and self-care, many women actively seek natural approaches to broaden their toolkit and repertoire of methods and medicines to address their diverse health needs. NHPs play an important role in this context, with 51% of menopausal women globally using them (32).

Integrating NHPs with conventional medical practice can offer women more holistic and personalized care options, especially where conventional options may be limited or have undesirable side effects (33). However, most commonly, self-prescribed medications are not always the most effective and/or best indicated. In Australia, a study of 2,020 women, over half of whom were postmenopausal, identified phytoestrogens (e.g., red clover and soy supplements), evening primrose oil, ginseng, and black cohosh as the most commonly used NHPs for hot flushes, whereas omega-3 supplements and glucosamine were most frequently used for other symptoms (e.g., CVD, joint pain). A majority of Australian women are thus using NHPs that are either known to be ineffective for their intended indication or whose efficacy and long-term safety have not yet been established (34). Therefore, the effective and safe use of NHPs alongside conventional options requires systematic frameworks that can generate robust scientific evidence on NHPs. Such evidence should encompass the pharmacological and biochemical actions of NHPs, including their biological targets and mechanisms. While some NHPs have been studied in clinical trials, many have not been evaluated with the same rigor as conventional medicines. Communication of the scientific foundations and validation of NHP use must also be enhanced.

In response to these needs and to inform a more systematic approach to the development and integration of NHPs into clinical practice, a multidisciplinary expert group of clinician-scientists, researchers, a pharmacist, and naturopaths gathered in Australia in February 2024. During this meeting, experts exchanged views and discussed their experience with natural treatments and relevant clinical evidence. The group, whose members are also authors of this paper, focused on the complementary use of NHPs to support women’s health in midlife. Discussions were guided by a semi-structured agenda and informed by pre-circulated background materials. While no formal consensus-building steps were undertaken, the dialogue surfaced key knowledge gaps and areas of shared concern that warrant further attention.

This narrative review draws from the group’s discussions, supplemented by a content-led literature search (PubMed; includes publications available up to 9 December 2024) to outline key considerations requiring further exploration. No restrictions were placed on publication date or study type.

This article provides a structured overview of the practical, clinical, and regulatory considerations surrounding NHP use, from the authors’ perspective, and should not be perceived as professional guidance. To this end, we sought to identify key challenges and propose high-level strategies for future work. The intention is to encourage informed discussion among stakeholders and promote the responsible integration of NHPs into care pathways. We believe that this can be achieved by seeking the best available scientific evidence and linking these insights with traditional knowledge in a way that is meaningful to both scientific/medical and traditional systems of health knowledge.

In reframing these broader insights as practical considerations for action, we identified several notable barriers to the safe and effective integration of NHPs into clinical practice, informed by the group’s diverse perspectives and illustrated with examples from the literature. Depending on the specific jurisdiction or setting, the challenges may be related to regulatory systems, scientific evidence, preparedness of health professionals, and/or consumer behavior. To guide the discussion in the following sections, Table 1 presents a summary of the key barriers identified, their implications, and potential strategies to help address them.

**Table 1:** Summary of key barriers and potential strategies for the integration of NHPs into clinical practice.

Category	Barriers	Implications	Potential Strategies
Regulatory frameworks	Lack of standardized mechanisms, definitions, and classification systems for regulating traditional medicine practices and NHPs across jurisdictions	Hinders systematic oversight, consistent regulation, and integration of NHPs into healthcare systems	Develop updated research guidelines and systematic frameworks for regulation and oversight Establish consensus on NHP definitions and classifications across jurisdictions
Scientific evidence and formulation	Inconsistent or low-quality evidence on some NHPs’ efficacy and safety Differences in activity spectrum across extracts (e.g., root vs. leaf) Complex composition with multiple bioactive compounds and diverse actions Poor systemic bioavailability of some compounds (e.g., curcumin) Varied efficacy and safety across different chemical forms of the same ingredient	Cause skepticism among health professionals Undermine the ability to generate consistent and reliable efficacy/safety conclusions Hinder standardization of product quality and dosing	Conduct well-designed clinical trials and systematic reviews using rigorous methodologies Use defined, standardized extracts and doses; assess specific outcomes and include appropriate controls Apply drug delivery technologies (e.g., nanoparticles, bioenhancers) to improve bioavailability where needed Systematically evaluate different chemical forms and their physiological effects
Health professionals	Health professionals have limited training and confidence to advise on NHPs	Limits ability to provide guidance during patient consultations	Include NHPs in curricula and continuing education
Consumers	Use of NHPs alongside conventional medicines without disclosure and monitoring by health professionals, particularly among older adults Exposure to conflicting or inaccurate information, especially from online sources	Increased risk of adverse effects and herb-drug interactions Confusion and misuse due to unreliable or misleading information	Foster open provider-patient communication and routine inquiry about NHP use Educate consumers on safe use of NHPs Develop accessible, evidence-based communication tools (e.g., traffic light system by AMS)

NHPs, Natural health products; AMS, Australasian Menopause Society.

## 2 Adapting systematic frameworks for evaluating efficacy and safety of natural health products

Although knowledge of NHPs as used in traditional medicine can inform attempts to study and integrate them into conventional care, a fundamentally evidence-based approach is critical. Despite their popularity and potential benefits based on accounts from traditional practice, the scientific evidence supporting the efficacy and safety of NHPs is often inconsistent or of suboptimal quality (34–36). This can lead to hesitancy and skepticism among health professionals regarding using NHPs, even where reasonable evidence may be available.

A significant obstacle at the systems level is the lack of standardized mechanisms and policy frameworks for regulating, overseeing, and ensuring the safety, quality, and effectiveness of traditional medicine practices, practitioners, and products (1, 33). The last extensive global guidance from the World Health Organization (WHO) on research and evaluation concerning the safety, efficacy, and quality of NHPs was issued more than two decades ago in 2000 (37). Updated research guidelines are urgently needed. Regulation of NHPs is far less stringent than that of conventional medicines. Globally, there is no consensus on the definitions, terminologies, and classifications of NHPs, leading to variations in how they are regulated (1). Systematic frameworks that effectively link traditional knowledge with scientific insights are needed if we are to better integrate the use of NHPs with conventional care to support women’s health in midlife. Rigorous evaluation of NHPs, mirroring the approach taken with conventional medicines, is crucial for establishing safety and efficacy. This will pave the way for the responsible integration of NHPs into patient care, wherever they are adequately supported by evidence.

Unlike synthetic drugs with a single, well-defined active ingredient, NHPs, such as ashwagandha and curcumin, are typically complex mixtures of multiple bioactive compounds with diverse bodily targets and actions (38, 39). This complexity can lead to challenges in standardizing efficacy and safety assessments, and thus, not all the regulatory standards required for conventional drugs may apply to NHPs. However, we propose adopting several key elements of Health Technology Assessment (HTA) frameworks (40), with appropriate modifications that consider the complexity of NHPs to systematically identify, assess, and evaluate products with promising therapeutic potential.

As highlighted by the WHO, “fundamental to implementing an integrated health system is acknowledging and bringing together different ways of knowing and/or different forms of evidence” (33). The development and integration of NHPs within healthcare should be grounded in systematic methods, particularly measurement of valid clinical outcomes, but can certainly be informed by known traditional uses. This knowledge generation and synthesis process should be accompanied by the creation/optimization of robust databases to catalog NHPs and systematically document their therapeutic effects. These databases would support further annotation through comprehensive literature reviews and/or insights from experts who regularly use them (including, but not necessarily limited to, naturopaths). Systematic evaluation of all the existing evidence is essential to reach evidence-based conclusions regarding the efficacy and safety of any NHP. Ideally, such resources would facilitate identifying active compounds, elucidating their mechanisms of action at the molecular level, and validating their efficacy and safety through controlled clinical studies. The databases should be updated regularly to incorporate new findings and ensure their ongoing relevance in the field (41).

## 3 Key considerations

In implementing frameworks to enhance the systematic identification, assessment, and evaluation of NHPs, we identified three key considerations: (1) Differences in the activity spectrum of extracts (e.g., whole-plant extracts vs. different plant parts), (2) bioavailability of active compounds, and (3) the chemical form(s) of the product. Each of these may significantly influence the therapeutic and/or safety profile of an NHP, including how its components work together and interact with other substances. We discuss these considerations using examples of NHPs that have gained interest from consumers and health professionals for reasons such as suitability for a range of health needs, coupled with a relatively safe profile and few significant drug interactions.

### 3.1 Differences in the activity spectrum of extracts from different plant parts

One such example is ashwagandha (*Withania somnifera*), which has been traditionally used for over 2,500 years for its benefits on various physiological systems (42, 43). It is currently marketed for enhancing stress management, cognitive abilities, and physical performance (43, 44). The ashwagandha plant contains a diverse array of phytochemicals, with approximately 140 distinct types identified—including withanolides, withanosides, sitoindosides, steroidal lactones, and alkaloids—that offer a broad spectrum of therapeutic benefits (43–45). The specific concentration and ratio of these compounds in extracts can vary considerably depending on the plant part used (roots vs. leaves), the cultivation conditions, and processing methods (46). Ashwagandha root extracts are primarily reported to have adaptogenic, anti-inflammatory, and neuroprotective properties, which are attributed to high concentrations of withaferin A and withanolide D. Leaf extracts, with different compositions and ratios of active compounds, reportedly have anti-inflammatory properties (47). Given the considerable variation in ashwagandha supplement compositions on the market, ranging from root-only extracts to those including leaf components (48), there is a clear need to systematically study and characterize different types of extracts and their biological activities.

Addressing the complexity of NHPs such as ashwagandha requires systematic studies using defined, standardized extracts and doses of specific plant parts. Studies should assess specific outcomes and/or biomarkers and include appropriate controls to enhance the reliability and comparability of research findings. For example, a recent study assessing the efficacy and tolerability of ashwagandha on menopausal transition symptoms, such as mood swings, sleep disturbance, and increased inflammation, may serve as a model (49). Using standardized extracts and doses (300 mg of ashwagandha root extract twice daily), the study measured outcomes with relevant and specific biomarkers, including the menopause rating scale and menopause-specific quality of life questionnaires, and hormonal parameters (E2, FSH, LH, testosterone) (49). The findings indicated significant improvements in menopausal symptoms and hormonal profiles compared with the placebo group. Using a placebo control helped ensure the improvements could be attributed to the use of ashwagandha root extract. To effectively utilize ashwagandha or any other NHP with promising potential for midlife health, well-designed, targeted clinical trials are needed to evaluate their effects and safety thoroughly. It is also important to standardize the methods of cultivation, harvesting, and processing to ensure consistent product quality.

### 3.2 Bioavailability of active compounds

Curcumin, a bioactive compound found in turmeric (*Curcuma longa*), serves as an excellent example of how low bioavailability may limit the therapeutic use of NHPs (50, 51). Curcumin is known for its anti-inflammatory and antioxidant benefits, which contribute to its use for a diverse range of illnesses (50, 51). Its effects on mitigating oxidative stress and improving pain and physical function have also been widely reported (52–55). However, the poor systemic bioavailability of curcumin makes effective dosing difficult, contributing to inconsistent clinical outcomes (56, 57). Its low bioavailability often means that the compound does not reach the target tissues in sufficient quantities, necessitating higher doses that may lead to side effects or interactions with other medications (51, 56).

Advancements in drug delivery systems have significantly improved the bioavailability of conventional medicines and can be applied to NHPs like curcumin. Examples include nanotechnology-based systems (e.g., liposomes, nanoparticles) that enhance pharmacokinetics and drug efficacy, or phytosomes that increase oral bioavailability by facilitating the passage of compounds through lipid membranes (50, 58). Additionally, bioenhancers that modulate drug metabolism can be incorporated to increase the absorption and availability of active compounds (58). For curcumin, advances such as the use of the bioenhancer piperine, nanoparticulate systems, emulsions, phospholipid complexes, liposomal carriers, polymeric micelles, and synthetic analogs have helped address bioavailability, absorption, and tissue targeting challenges (58, 59). Nevertheless, further research is needed to fully evaluate and optimize the pharmacokinetic effects and medicinal potential of these innovative curcumin formulations in clinical trials. This will allow the identification of effective administration methods and optimal dose ranges within populations.

### 3.3 Differences between chemical forms of ingredients

The US Food and Drug Administration (FDA) and European Medicines Agency (EMA) acknowledge that different salts, esters, or non-covalent derivatives of the same active moiety may exhibit different efficacy and/or safety properties in conventional drug products (60, 61). This principle also applies to NHPs, as illustrated by the various forms of magnesium, which differ in their chemical composition, solubility, bioavailability, and interactions with other molecules, thereby affecting their target and function (62). Magnesium, a vital mineral, contributes to several metabolic processes and is essential for the production of mitochondrial energy, as well as optimal brain, heart, and skeletal muscle function. Interestingly, its distribution in the body can be influenced by estrogen (63, 64).

Magnesium aspartate, oxide, hydroxide, and citrate are all common salt forms used in oral supplements, but magnesium citrate, aspartate, and other organic salt forms show much higher bioavailability than magnesium oxide (63–65). Today, magnesium hydroxide (milk of magnesia) and magnesium oxide are commonly used as laxatives. Other forms of magnesium supplements reported to have specific effects include magnesium glycinate (enhancing sleep quality), magnesium taurate (cardiovascular health), and magnesium L-threonate, which can cross the blood–brain barrier and is reported to improve memory and cognitive function (63–65). The differences between various chemical forms and physiological effects of magnesium supplements are important and should be considered in studies seeking to determine which magnesium salts and dosage forms are most effective for specific health outcomes. This understanding is critical for developing and producing efficacious supplements.

## 4 Ensuring the safe use of natural health products

It is essential for health professionals and consumers to be well-informed about the safe and effective use of NHPs alone or in conjunction with conventional medicines to maximize therapeutic benefits and minimize potential risks. For example, reports indicate that consumers continue to use certain NHPs, like black cohosh (*Actaea racemosa*), to alleviate menopausal symptoms without professional guidance, despite inconsistent evidence regarding their safety, including potential hepatotoxic effects (34, 66–68). A significant number of individuals reportedly do not disclose NHP use to their doctors, which is a concern, as not all health professionals may initiate discussions about NHP usage during consultations (8). Common reasons for nondisclosure include the belief that NHPs are inherently safe because they are “natural” and the perception that such use is irrelevant to their conventional treatment (8).

Additionally, many people use NHPs alongside over-the-counter and/or prescription medicines. Over half of Australian adults who use NHPs report concurrent use with other medications on the same day. This practice is five times more common among individuals over 65 years old compared with young adults (18–24 years old) (5). This potentially leads to herb-drug interactions that can compromise the efficacy and safety of conventional treatments (38, 69). One emerging example is berberine, a naturally occurring alkaloid utilized in traditional Chinese and Ayurvedic medicine for over 3,000 years. Potential health benefits attributed to berberine include blood sugar control, cholesterol management, cardiovascular support, weight loss promotion, and inflammation reduction (70, 71). Standard doses of berberine are usually well-tolerated (72); however, berberine can affect the metabolism of certain common drugs, including cyclosporine, anticoagulants, diabetes and blood pressure drugs, and sedatives (70, 72). Berberine interacts with cytochrome CYP3A4 and intestinal P-glycoprotein, significantly affecting the bioavailability of orally administered medications (72). Given the growing interest in berberine as a potentially beneficial NHP, due to its hypoglycemic and anti-inflammatory effects (73–76), optimizing patient safety necessitates ensuring its well-informed co-administration with conventional treatments.

## 5 Complementary use of natural health products

NHPs have potential as complementary or adjunctive therapies alongside conventional medicines in a variety of settings. For instance, palmitoylethanolamide (PEA) is an NHP with promising potential as a complementary option for pain management. As a member of the N-acyl-ethanolamine family, PEA is a bioactive lipid synthesized in response to cellular injury and is found in all body tissues, including the brain (77). PEA has anti-inflammatory, analgesic, anticonvulsant, and neuroprotective effects (77). In managing chronic pain—a common issue among midlife women—PEA is well-suited to be used adjunctively as it promotes endogenous cannabinoid system activity through its ability to augment the activity of cannabinoid receptors CB1 and CB2. These receptors are expressed in nerve tissue and immune cells and help modulate the inflammatory response by suppressing immune cell activation, which can reduce pain perception (78). PEA has consistently shown activity in placebo- or active-controlled studies (79–82). In a double-blind, placebo-controlled trial involving 636 sciatic pain patients, those receiving PEA treatment (600 mg/day) reported a reduction in subjective pain scores from 7.1/10 to 2.1/10 compared to the placebo group (83). Moreover, numerous studies also associate PEA with enhanced well-being and functional ability, noting minimal reported side effects (79–82).

Other NHPs have also shown promise as complementary treatments for managing milder symptoms alongside conventional medicines (52–55, 84, 85). Systematic analyses indicate that curcumin has effects on arthritis pain and function comparable to non-steroidal anti-inflammatory drugs (NSAIDs), modulating the NF-κB immune response similarly and without significant adverse events (52–55). Oleoresins from *Boswellia serrata* and other species have long been used in traditional medicine, and the major active components, the boswellic acids, have been shown to have anti-inflammatory properties (86, 87). Boswellia is marketed for various purposes, including managing mild pain and other symptoms in people with conditions such as osteoarthritis. Similar to curcumin, systematic analyses indicate that Boswellia is more effective than placebo in providing pain relief and improving functional outcomes in people with osteoarthritis while maintaining a favorable safety profile (55, 84, 85). Current evidence suggests that formulations containing curcumin and Boswellia extracts could be beneficial for mild pain and inflammation-related symptoms.

For many NHPs, no definitive conclusions are available regarding their efficacy or effectiveness due to the paucity and/or low quality of available evidence (44, 55, 80, 84, 88–91). While considering the unique features and complexity of NHPs, as outlined above, future studies should implement rigorous methodologies to reduce bias. These include randomization, allocation concealment, managing dropouts, and avoidance of selective reporting. Adequate attention must also be given to studying side effects as well as benefits. Such investment in systematic evidence generation will support safer and more effective use of NHPs.

The use of NHPs should be guided by health professionals. However, professionals often struggle to keep up with new evidence that may be equivocal or of uncertain quality; regulatory standards are of limited use in terms of practical guidance (92). Consumers are faced with vast amounts of conflicting or inaccurate information online and on social media, leading to confusion and potential misuse of NHPs, as these platforms are often unregulated in many countries, lacking oversight such as expert review or fact-checking (93–95). Enhanced educational resources and improved communication strategies for professionals and consumers are essential to promoting the safe and informed use of NHPs. While robust evidence is essential for credibility, accurate and effective communication of this information to both professionals and consumers will help build trust and confidence in integrating NHPs in areas such as menopausal health.

A comprehensive training curriculum for health professionals that provides a balanced view of safety, benefits, and appropriate applications of NHPs would support informed discussions during health consultations. A cross-sectional online survey of 2,341 pharmacists from nine countries highlighted the urgent need to incorporate NHP education into undergraduate curricula and emphasized the importance of continuing education courses and workshops for trainees and practicing professionals to stay updated on the latest research and practice trends (96). Making educational materials and resources accessible to people with different general and health literacy levels is another priority (41). Enhancing health literacy can empower individuals to make informed choices and engage in self-care practices involving NHPs. For instance, although graphical presentations of a systematic analysis of dietary supplements for osteoarthritis exist, these are primarily intended for health professionals (97). The information could be adapted into a more consumer-friendly infographic to better communicate these important findings to a broad audience. A good example is the Australasian Menopause Society’s (AMS) guide on NHPs for managing menopausal symptoms. The guide uses an intuitive traffic light system to present current evidence-based recommendations, making the information easy to interpret. For instance, AMS highlights that some products, like red clover, evening primrose oil, black cohosh, and omega-3 supplements, require more research. This approach makes it easier for consumers to understand their options and encourages a cautious yet open-minded approach to their use in clinical practice (66).

This article is a narrative review informed by multidisciplinary expert discussions and supplemented by a thematic literature search. As such, we acknowledge inherent limitations in the scope and completeness of the selection of sources. While we aimed to provide a balanced overview of key considerations on the integration of NHPs into clinical practice, not all recommendations may be generalizable across regulatory or cultural settings due to variations in healthcare systems, practice norms, and product availability. These limitations underscore the need for further region-specific and systematic investigations to complement the perspectives offered in this review.

## 6 Conclusion

While NHPs are increasingly considered relevant by midlife women and other consumers due to their perceived benefits based on traditional use, many NHPs lack rigorous scientific validation. With rising global consumption and the increasingly common use of NHPs alongside conventional healthcare, appropriate systematic evaluation frameworks are urgently needed. To integrate NHPs effectively and safely within our care systems, we need to draw upon evidence generation and dissemination methodologies parallel to those used for conventional medicines but tailored to address the unique complexities of NHPs. Safe, responsible, and informed use of NHPs will also require enhanced continuing education for health professionals and improved consumer communication strategies. Future work should support these efforts by building the evidence base and aligning practice, education, and policy. To enhance patient health outcomes and realize the potential of NHPs, we must have the evidence we need and ensure that all stakeholders understand how best to use it.

## References

[1] Thakkar S Anklam E Xu A Ulberth F Li J Li B. Regulatory landscape of dietary supplements and herbal medicines from a global perspective. Regul Toxicol Pharmacol. (2020) 114:104647. doi: 10.1016/j.yrtph.2020.104647, PMID: 32305367

[2] Dwyer JT Coates PM Smith MJ. Dietary supplements: regulatory challenges and research resources. Nutrients. (2018) 10:41. doi: 10.3390/nu10010041, PMID: 29300341 PMC5793269

[3] Djaoudene O Romano A Bradai YD Zebiri F Ouchene A Yousfi Y. A global overview of dietary supplements: regulation, market trends, usage during the Covid-19 pandemic, and health effects. Nutrients. (2023) 15:3320. doi: 10.3390/nu15153320, PMID: 37571258 PMC10421343

[4] Therapeutic Goods Administration (TGA). Complementary medicines: Australian Government Department of Health and Aged Care. Available online at: https://www.tga.gov.au/topics/complementary-medicines (Accessed June 3, 2024).

[5] Harnett J McIntyre E Adams J Addison T Bannerman H Egelton L. Prevalence and characteristics of Australians complementary medicine product use, and concurrent use with prescription and over-the-counter medications-a cross sectional study. Nutrients. (2023) 15:327. doi: 10.3390/nu15020327, PMID: 36678198 PMC9860983

[6] Tangkiatkumjai M Boardman H Walker DM. Potential factors that influence usage of complementary and alternative medicine worldwide: a systematic review. BMC Complement Med Ther. (2020) 20:363. doi: 10.1186/s12906-020-03157-2, PMID: 33228697 PMC7686746

[7] Reid R Steel A Wardle J Trubody A Adams J. Complementary medicine use by the Australian population: a critical mixed studies systematic review of utilisation, perceptions and factors associated with use. BMC Complement Altern Med. (2016) 16:176. doi: 10.1186/s12906-016-1143-8, PMID: 27289517 PMC4902999

[8] Harnett JE McIntyre E Steel A Foley H Sibbritt D Adams J. Use of complementary medicine products: a nationally representative cross-sectional survey of 2019 Australian adults. BMJ Open. (2019) 9:e024198. doi: 10.1136/bmjopen-2018-024198, PMID: 31315853 PMC6661602

[9] van Dijk GM Kavousi M Troup J Franco OH. Health issues for menopausal women: the top 11 conditions have common solutions. Maturitas. (2015) 80:24–30. doi: 10.1016/j.maturitas.2014.09.013, PMID: 25449663

[10] Jaspers L Daan NM van Dijk GM Gazibara T Muka T Wen KX. Health in middle-aged and elderly women: a conceptual framework for healthy menopause. Maturitas. (2015) 81:93–8. doi: 10.1016/j.maturitas.2015.02.010, PMID: 25813865

[11] World Health Organization. Menopause: World Health Organization. Available online at: https://www.who.int/news-room/fact-sheets/detail/menopause (Accessed May 30, 2024).

[12] Harlow SD Derby CA. Women's midlife health: why the midlife matters. Womens Midlife Health. (2015) 1:5. doi: 10.1186/s40695-015-0006-7, PMID: 30766692 PMC6214213

[13] Talaulikar V. Menopause transition: physiology and symptoms. Best Pract Res Clin Obstet Gynaecol. (2022) 81:3–7. doi: 10.1016/j.bpobgyn.2022.03.003, PMID: 35382992

[14] Hoga L Rodolpho J Gonçalves B Quirino B. Women's experience of menopause: a systematic review of qualitative evidence. JBI Database Syst Rev Implement Rep. (2015) 13:250–337. doi: 10.11124/jbisrir-2015-1948, PMID: 26455946

[15] Santoro N Roeca C Peters BA Neal-Perry G. The menopause transition: signs, symptoms, and management options. J Clin Endocrinol Metab. (2021) 106:1–15. doi: 10.1210/clinem/dgaa764, PMID: 33095879

[16] Palacios S Chedraui P Sánchez-Borrego R Coronado P Nappi RE. Obesity and menopause. Gynecol Endocrinol. (2024) 40:2312885. doi: 10.1080/09513590.2024.2312885, PMID: 38343134

[17] Rathnayake N Rathnayake H Lekamwasam S. Age-related trends in body composition among women aged 20-80 years: a cross-sectional study. J Obes. (2022) 2022:4767793. doi: 10.1155/2022/4767793, PMID: 35154825 PMC8828324

[18] Madsen TE Sobel T Negash S Shrout Allen T Stefanick ML Manson JE. A review of hormone and non-hormonal therapy options for the treatment of menopause. Int J Women's Health. (2023) 15:825–36. doi: 10.2147/ijwh.S379808, PMID: 37255734 PMC10226543

[19] Ji MX Yu Q. Primary osteoporosis in postmenopausal women. Chronic Dis Transl Med. (2015) 1:9–13. doi: 10.1016/j.cdtm.2015.02.006, PMID: 29062981 PMC5643776

[20] Rodriguez YA Abramson BL. Cardiovascular and physiological risk factors in women at midlife and beyond. Can J Physiol Pharmacol. (2024) 102:442–451. doi: 10.1139/cjpp-2023-0468 38739947

[21] Thurston RC Chang Y Kline CE Swanson LM El Khoudary SR Jackson EA. Trajectories of sleep over midlife and incident cardiovascular disease events in the study of women’s health across the nation. Circulation. (2024) 149:545–55. doi: 10.1161/CIRCULATIONAHA.123.066491, PMID: 38284249 PMC10922947

[22] Gibson CJ Li Y Bertenthal D Huang AJ Seal KH. Menopause symptoms and chronic pain in a national sample of midlife women veterans. Menopause. (2019) 26:708–13. doi: 10.1097/gme.0000000000001312, PMID: 30839364

[23] Bromberger JT Kravitz HM Chang YF Cyranowski JM Brown C Matthews KA. Major depression during and after the menopausal transition: study of women's health across the nation (swan). Psychol Med. (2011) 41:1879–88. doi: 10.1017/S003329171100016X, PMID: 21306662 PMC3584692

[24] Vivian-Taylor J Hickey M. Menopause and depression: is there a link? Maturitas. (2014) 79:142–6. doi: 10.1016/j.maturitas.2014.05.014, PMID: 24951102

[25] Kulkarni J Gurvich C Mu E Molloy G Lovell S Mansberg G. Menopause depression: under recognised and poorly treated. Aust N Z J Psychiatry. (2024) 58:636–40 20240518. doi: 10.1177/00048674241253944, PMID: 38761367 PMC11308326

[26] El Khoudary SR Greendale G Crawford SL Avis NE Brooks MM Thurston RC. The menopause transition and women's health at midlife: a progress report from the study of women's health across the nation (swan). Menopause. (2019) 26:1213–27. doi: 10.1097/gme.0000000000001424, PMID: 31568098 PMC6784846

[27] Conde DM Verdade RC Valadares ALR Mella LFB Pedro AO Costa-Paiva L. Menopause and cognitive impairment: a narrative review of current knowledge. World J Psychiatry. (2021) 11:412–28. doi: 10.5498/wjp.v11.i8.412, PMID: 34513605 PMC8394691

[28] The Australasian Menopause Society (AMS). What is menopausal hormone therapy (MHT) and is it safe? Available online at: https://www.menopause.org.au/health-info/fact-sheets/what-is-menopausal-hormone-therapy-mht-and-is-it-safe (Accessed September 6, 2024).

[29] Minkin MJ. Menopause: hormones, lifestyle, and optimizing aging. Obstet Gynecol Clin N Am. (2019) 46:501–14. doi: 10.1016/j.ogc.2019.04.008, PMID: 31378291

[30] Davis SR Magraith K. Advancing menopause care in Australia: barriers and opportunities. Med J Aust. (2023) 218:500–2. doi: 10.5694/mja2.51981, PMID: 37330995

[31] Cagnacci A Venier M. The controversial history of hormone replacement therapy. Medicina (Kaunas). (2019) 55:602. doi: 10.3390/medicina55090602, PMID: 31540401 PMC6780820

[32] Posadzki P Lee MS Moon TW Choi TY Park TY Ernst E. Prevalence of complementary and alternative medicine (CAM) use by menopausal women: a systematic review of surveys. Maturitas. (2013) 75:34–43. doi: 10.1016/j.maturitas.2013.02.005, PMID: 23497959

[33] World Health Organization. Integrating traditional and complementary medicine into health systems: social, economic and health considerations Geneva: World Health Organization (2023).

[34] Gartoulla P Davis SR Worsley R Bell RJ. Use of complementary and alternative medicines for menopausal symptoms in Australian women aged 40-65 years. Med J Aust. (2015) 203:146, 146e.1. doi: 10.5694/mja14.01723, PMID: 26224187

[35] Moses G. What's in complementary medicines? Aust Prescr. (2019) 42:82–3. doi: 10.18773/austprescr.2019.024, PMID: 31363302 PMC6594845

[36] Johnson A Roberts L Elkins G. Complementary and alternative medicine for menopause. J Evid Based Integr Med. (2019) 24:2515690x19829380. doi: 10.1177/2515690x19829380, PMID: 30868921 PMC6419242

[37] World Health Organization. General guidelines for methodologies on research and evaluation of traditional medicine Geneva: World Health Organization (2000).

[38] Pelkonen O Xu Q Fan T-P. Why is research on herbal medicinal products important and how can we improve its quality? J Tradit Complement Med. (2014) 4:1–7. doi: 10.4103/2225-4110.124323, PMID: 24872927 PMC4032837

[39] Nasim N Sandeep IS Mohanty S. Plant-derived natural products for drug discovery: current approaches and prospects. Nucleus. (2022) 65:399–411. doi: 10.1007/s13237-022-00405-3, PMID: 36276225 PMC9579558

[40] World Health Organization. Health technology assessment: World Health Organization. Available online at: https://www.who.int/health-topics/health-technology-assessment (Accessed July 9, 2024).

[41] World Health Organization. Who traditional medicine strategy: 2014–2023 Geneva: World Health Organization (2013).

[42] Joshi VK Joshi A. Rational use of Ashwagandha in Ayurveda (traditional Indian medicine) for health and healing. J Ethnopharmacol. (2021) 276:114101. doi: 10.1016/j.jep.2021.114101, PMID: 33831467

[43] Mikulska P Malinowska M Ignacyk M Szustowski P Nowak J Pesta K. Ashwagandha (*Withania Somnifera*)—current research on the health-promoting activities: a narrative review. Pharmaceutics. (2023) 15:1057. doi: 10.3390/pharmaceutics15041057, PMID: 37111543 PMC10147008

[44] Della Porta M Maier JA Cazzola R. Effects of *Withania Somnifera* on cortisol levels in stressed human subjects: a systematic review. Nutrients. (2023) 15:5015. doi: 10.3390/nu15245015, PMID: 38140274 PMC10745833

[45] Bashir A Nabi M Tabassum N Afzal S Ayoub M. An updated review on phytochemistry and molecular targets of *Withania Somnifera* (L.) Dunal (Ashwagandha). Front Pharmacol. (2023) 14:14. doi: 10.3389/fphar.2023.1049334, PMID: 37063285 PMC10090468

[46] Saleem S Muhammad G Hussain MA Altaf M Bukhari SNA. *Withania Somnifera* L.: insights into the phytochemical profile, therapeutic potential, clinical trials, and future prospective. Iran J Basic Med Sci. (2020) 23:1501–26. doi: 10.22038/ijbms.2020.44254.10378, PMID: 33489024 PMC7811807

[47] Mahmoudi R Ansari S Haghighizadeh MH Maram NS Montazeri S. Investigation the effect of jujube seed capsule on sleep quality of postmenopausal women: a double-blind randomized clinical trial. Biomedicine (Taipei). (2020) 10:42–8. doi: 10.37796/2211-8039.1038, PMID: 33854934 PMC7735973

[48] Mukherjee PK Banerjee S Biswas S Das B Kar A Katiyar CK. *Withania somnifera* (L.) Dunal - modern perspectives of an ancient rasayana from Ayurveda. J Ethnopharmacol. (2021) 264:113157. doi: 10.1016/j.jep.2020.113157, PMID: 32783987

[49] Gopal S Ajgaonkar A Kanchi P Kaundinya A Thakare V Chauhan S. Effect of an ashwagandha (*Withania somnifera*) root extract on climacteric symptoms in women during perimenopause: a randomized, double-blind, placebo-controlled study. J Obstet Gynaecol Res. (2021) 47:4414–25. doi: 10.1111/jog.15030, PMID: 34553463

[50] Sharifi-Rad J Rayess YE Rizk AA Sadaka C Zgheib R Zam W. Turmeric and its major compound curcumin on health: bioactive effects and safety profiles for food, pharmaceutical, biotechnological and medicinal applications. Front Pharmacol. (2020) 11:01021. doi: 10.3389/fphar.2020.01021, PMID: 33041781 PMC7522354

[51] Abd El-Hack ME El-Saadony MT Swelum AA Arif M Abo Ghanima MM Shukry M. Curcumin, the active substance of turmeric: its effects on health and ways to improve its bioavailability. J Sci Food Agric. (2021) 101:5747–62. doi: 10.1002/jsfa.11372, PMID: 34143894

[52] Paultre K Cade W Hernandez D Reynolds J Greif D Best TM. Therapeutic effects of turmeric or curcumin extract on pain and function for individuals with knee osteoarthritis: a systematic review. BMJ Open Sport Exerc Med. (2021) 7:e000935. doi: 10.1136/bmjsem-2020-000935, PMID: 33500785 PMC7812094

[53] Kou H Huang L Jin M He Q Zhang R Ma J. Effect of curcumin on rheumatoid arthritis: a systematic review and meta-analysis. Front Immunol. (2023) 14:1121655. doi: 10.3389/fimmu.2023.1121655, PMID: 37325651 PMC10264675

[54] Zeng L Yang T Yang K Yu G Li J Xiang W. Efficacy and safety of curcumin and *Curcuma Longa* extract in the treatment of arthritis: a systematic review and meta-analysis of randomized controlled trial. Front Immunol. (2022) 13:891822. doi: 10.3389/fimmu.2022.891822, PMID: 35935936 PMC9353077

[55] Liu X Machado GC Eyles JP Ravi V Hunter DJ. Dietary supplements for treating osteoarthritis: a systematic review and meta-analysis. Br J Sports Med. (2018) 52:167–75. doi: 10.1136/bjsports-2016-097333, PMID: 29018060

[56] El-Saadony MT Yang T Korma SA Sitohy M Abd El-Mageed TA Selim S. Impacts of turmeric and its principal bioactive curcumin on human health: pharmaceutical, medicinal, and food applications: a comprehensive review. Front Nutr. (2022) 9:1040259. doi: 10.3389/fnut.2022.1040259, PMID: 36712505 PMC9881416

[57] Shen L Liu CC An CY Ji HF. How does curcumin work with poor bioavailability? Clues from experimental and theoretical studies. Sci Rep. (2016) 6:20872. doi: 10.1038/srep20872, PMID: 26887346 PMC4757858

[58] Kesarwani K Gupta R Mukerjee A. Bioavailability enhancers of herbal origin: an overview. Asian Pac J Trop Biomed. (2013) 3:253–66. doi: 10.1016/s2221-1691(13)60060-x, PMID: 23620848 PMC3634921

[59] Olotu F Agoni C Soremekun O Soliman MES. An update on the pharmacological usage of curcumin: has it failed in the drug discovery pipeline? Cell Biochem Biophys. (2020) 78:267–89. doi: 10.1007/s12013-020-00922-5, PMID: 32504356

[60] U.S. Food and Drug Administration. Approved drug products with therapeutic equivalence evaluations | Orange book (2024). Available online at: https://www.fda.gov/drugs/development-approval-process-drugs/orange-book-preface (Accessed June 12, 2024).

[61] European Medicines Agency. Guideline on the investigation of bioequivalence Amsterdam: European Medicines Agency (2010). 10.1111/j.1742-7843.2009.00518.x 20070293

[62] Pardo MR Garicano Vilar E San Mauro Martín I Camina Martín MA. Bioavailability of magnesium food supplements: a systematic review. Nutrition. (2021) 89:111294. doi: 10.1016/j.nut.2021.111294, PMID: 34111673

[63] Blancquaert L Vervaet C Derave W. Predicting and testing bioavailability of magnesium supplements. Nutrients. (2019) 11:1663. doi: 10.3390/nu11071663, PMID: 31330811 PMC6683096

[64] Fiorentini D Cappadone C Farruggia G Prata C. Magnesium: biochemistry, nutrition, detection, and social impact of diseases linked to its deficiency. Nutrients. (2021) 13:1136. doi: 10.3390/nu13041136, PMID: 33808247 PMC8065437

[65] Schwalfenberg GK Genuis SJ. The importance of magnesium in clinical healthcare. Scientifica (Cairo). (2017) 2017:1–14. doi: 10.1155/2017/4179326, PMID: 29093983 PMC5637834

[66] The Australasian Menopause Society (AMS). Complementary medicine options for menopausal symptoms Melbourne: Australasian Menopause Society (2018).

[67] Stournaras E Tziomalos K. Herbal medicine-related hepatotoxicity. World J Hepatol. (2015) 7:2189–93. doi: 10.4254/wjh.v7.i19.2189, PMID: 26380043 PMC4561772

[68] Teschke R. Black cohosh and suspected hepatotoxicity: inconsistencies, confounding variables, and prospective use of a diagnostic causality algorithm. A critical review. Menopause. (2010) 17:426–40. doi: 10.1097/gme.0b013e3181c5159c, PMID: 20216279

[69] Mortada EM. Evidence-based complementary and alternative medicine in current medical practice. Cureus. (2024) 16:e52041. doi: 10.7759/cureus.52041 38344508 PMC10857488

[70] Neag MA Mocan A Echeverría J Pop RM Bocsan CI Crişan G. Berberine: botanical occurrence, traditional uses, extraction methods, and relevance in cardiovascular, metabolic, hepatic, and renal disorders. Front Pharmacol. (2018) 9:557. doi: 10.3389/fphar.2018.00557, PMID: 30186157 PMC6111450

[71] Ye Y Liu X Wu N Han Y Wang J Yu Y. Efficacy and safety of Berberine alone for several metabolic disorders: a systematic review and meta-analysis of randomized clinical trials. Front Pharmacol. (2021) 12:653887. doi: 10.3389/fphar.2021.653887, PMID: 33981233 PMC8107691

[72] Caliceti C Rizzo P Cicero AF. Potential benefits of Berberine in the management of perimenopausal syndrome. Oxidative Med Cell Longev. (2015) 2015:723093. doi: 10.1155/2015/723093, PMID: 25785174 PMC4346702

[73] Liang Y Xu X Yin M Zhang Y Huang L Chen R. Effects of Berberine on blood glucose in patients with type 2 diabetes mellitus: a systematic literature review and a meta-analysis. Endocr J. (2019) 66:51–63. doi: 10.1507/endocrj.EJ18-0109, PMID: 30393248

[74] Nazari A Ghotbabadi ZR Kazemi KS Metghalchi Y Tavakoli R Rahimabadi RZ. The effect of Berberine supplementation on glycemic control and inflammatory biomarkers in metabolic disorders: an umbrella meta-analysis of randomized controlled trials. Clin Ther. (2024) 46:e64–72. doi: 10.1016/j.clinthera.2023.10.019, PMID: 38016844

[75] Ilyas Z Perna S Al-Thawadi S Alalwan TA Riva A Petrangolini G. The effect of Berberine on weight loss in order to prevent obesity: a systematic review. Biomed Pharmacother. (2020) 127:110137. doi: 10.1016/j.biopha.2020.110137, PMID: 32353823

[76] Xie W Su F Wang G Peng Z Xu Y Zhang Y. Glucose-lowering effect of Berberine on type 2 diabetes: a systematic review and meta-analysis. Front Pharmacol. (2022) 13:1015045. doi: 10.3389/fphar.2022.1015045, PMID: 36467075 PMC9709280

[77] Clayton P Hill M Bogoda N Subah S Venkatesh R. Palmitoylethanolamide: a natural compound for health management. Int J Mol Sci. (2021) 22:5305. doi: 10.3390/ijms22105305, PMID: 34069940 PMC8157570

[78] Lago-Fernandez A Zarzo-Arias S Jagerovic N Morales P. Relevance of peroxisome proliferator activated receptors in multitarget paradigm associated with the endocannabinoid system. Int J Mol Sci. (2021) 22:1001. doi: 10.3390/ijms22031001, PMID: 33498245 PMC7863932

[79] Artukoglu BB Beyer C Zuloff-Shani A Brener E Bloch MH. Efficacy of palmitoylethanolamide for pain: a meta-analysis. Pain Physician. (2017) 20:353–62. 28727699

[80] Lang-Illievich K Klivinyi C Lasser C Brenna CTA Szilagyi IS Bornemann-Cimenti H. Palmitoylethanolamide in the treatment of chronic pain: a systematic review and meta-analysis of double-blind randomized controlled trials. Nutrients. (2023) 15:1350. doi: 10.3390/nu15061350, PMID: 36986081 PMC10053226

[81] Scuteri D Guida F Boccella S Palazzo E Maione S Rodríguez-Landa JF. Effects of Palmitoylethanolamide (pea) on nociceptive, musculoskeletal and neuropathic pain: systematic review and meta-analysis of clinical evidence. Pharmaceutics. (2022) 14:1672. doi: 10.3390/pharmaceutics14081672, PMID: 36015298 PMC9414729

[82] Schweiger V Schievano C Martini A Polati L Del Balzo G Simari S. Extended treatment with micron-size oral Palmitoylethanolamide (pea) in chronic pain: a systematic review and Meta-analysis. Nutrients. (2024) 16:1653. doi: 10.3390/nu16111653, PMID: 38892586 PMC11174044

[83] Keppel Hesselink JM Kopsky DJ. Palmitoylethanolamide, a neutraceutical, in nerve compression syndromes: efficacy and safety in sciatic pain and carpal tunnel syndrome. J Pain Res. (2015) 8:729–34. doi: 10.2147/jpr.S93106, PMID: 26604814 PMC4631430

[84] Bannuru RR Osani MC Al-Eid F Wang C. Efficacy of curcumin and Boswellia for knee osteoarthritis: systematic review and meta-analysis. Semin Arthritis Rheum. (2018) 48:416–29. doi: 10.1016/j.semarthrit.2018.03.001, PMID: 29622343 PMC6131088

[85] Yu G Xiang W Zhang T Zeng L Yang K Li J. Effectiveness of Boswellia and Boswellia extract for osteoarthritis patients: a systematic review and meta-analysis. BMC Complement Med Ther. (2020) 20:225. doi: 10.1186/s12906-020-02985-6, PMID: 32680575 PMC7368679

[86] Siddiqui MZ. *Boswellia Serrata*, a potential antiinflammatory agent: an overview. Indian J Pharm Sci. (2011) 73:255–61. doi: 10.4103/0250-474x.93507, PMID: 22457547 PMC3309643

[87] Al-Yasiry AR Kiczorowska B. Frankincense--therapeutic properties. Postepy Hig Med Dosw (Online). (2016) 70:380–91. doi: 10.5604/17322693.1200553, PMID: 27117114

[88] Akhgarjand C Asoudeh F Bagheri A Kalantar Z Vahabi Z Shab-Bidar S. Does Ashwagandha supplementation have a beneficial effect on the management of anxiety and stress? A systematic review and meta-analysis of randomized controlled trials. Phytother Res. (2022) 36:4115–24. doi: 10.1002/ptr.7598, PMID: 36017529

[89] Arab A Rafie N Amani R Shirani F. The role of magnesium in sleep health: a systematic review of available literature. Biol Trace Elem Res. (2023) 201:121–8. doi: 10.1007/s12011-022-03162-1, PMID: 35184264

[90] Cheah KL Norhayati MN Husniati Yaacob L Abdul Rahman R. Effect of Ashwagandha (*Withania somnifera*) extract on sleep: a systematic review and meta-analysis. PLoS One. (2021) 16:e0257843. doi: 10.1371/journal.pone.0257843, PMID: 34559859 PMC8462692

[91] Groenendijk I van Delft M Versloot P van Loon LJC de Groot L. Impact of magnesium on bone health in older adults: a systematic review and meta-analysis. Bone. (2022) 154:116233. doi: 10.1016/j.bone.2021.116233, PMID: 34666201

[92] von Schoen-Angerer T Manchanda RK Lloyd I Wardle J Szöke J Benevides I. Traditional, complementary and integrative healthcare: global stakeholder perspective on WHO's current and future strategy. BMJ Glob Health. (2023) 8:e013150. doi: 10.1136/bmjgh-2023-013150, PMID: 38050407 PMC10693890

[93] Sansevere ME White JD. Quality assessment of online complementary and alternative medicine information resources relevant to cancer. Integr Cancer Ther. (2021) 20:15347354211066081. doi: 10.1177/15347354211066081, PMID: 34923872 PMC8725029

[94] Ng JY Nayeni M Gilotra K. Quality of complementary and alternative medicine information for type 2 diabetes: a cross-sectional survey and quality assessment of websites. BMC Complement Med Ther. (2021) 21:233. doi: 10.1186/s12906-021-03390-3, PMID: 34535126 PMC8447516

[95] Ng JY Raja M Tahir U Thakar H Balkaran SL. Assessing the quality of complementary, alternative, and integrative medicine website information for cancer: a cross-sectional survey and analysis. Eur J Integr Med. (2023) 64:102309. doi: 10.1016/j.eujim.2023.102309

[96] Harnett JE Desselle SP Fernandes MB Yao D Modun D Hallit S. Defining and supporting a professional role for pharmacists associated with traditional and complementary medicines: a cross-country survey of pharmacists. Front Pharmacol. (2023) 14:14. doi: 10.3389/fphar.2023.1215475, PMID: 37654614 PMC10467277

[97] Liu X Eyles J McLachlan AJ Mobasheri A. Which supplements can I recommend to my osteoarthritis patients? Rheumatology. (2018) 57:iv75–87. doi: 10.1093/rheumatology/key005, PMID: 29506080

